# 

**Published:** 1913-08

**Authors:** 


### CONTENTS.

ORIGINAL COMMUNICATIONS—

Spectomy with Report of a Case. By E. C. Davis, M. D., Atlanta, Ga. .......... 191

Summer Diarrhoea. By C. O. Williams, M. D., West Point, Ga. .......... 194

The Radiograph in Relation to the Diagnosis and Treatment of Gastric and Intestinal Lesions. By H. P. Cole, M. D., Mobile, Ala. .......... 197

The Treatment of Syphilis. By Edgar G. Ballenger, M. D., and Omar F. Elder, Atlanta, Ga. .......... 203

EDITORIAL .......... 207

SELECTIONS AND ABSTRACTS .......... 213

BOOK REVIEWS .......... 232

